# Efficacy and safety of bempedoic acid alone or combining with other lipid-lowering therapies in hypercholesterolemic patients: a meta-analysis of randomized controlled trials

**DOI:** 10.1186/s40360-020-00463-w

**Published:** 2020-12-04

**Authors:** Xiang Zhao, Xubiao Ma, Xing Luo, Zhihua Shi, Ziwen Deng, Yuanxiang Jin, Zhipeng Xiao, Liming Tan, Pingfang Liu, Shilong Jiang, Yuanglu Shu, Bing Tang, Chengfeng Qiu

**Affiliations:** 1 Department of General Practice, The First People’s Hospital of Huaihua, University of South China, Huaihua, 418000 People’s Republic of China; 2 Evidence-based Medicine and Clinical Center, The First People’s Hospital of Huaihua, University of South China, Huaihua, 418000 People’s Republic of China; 3 Department of Pharmacology, The First People’s Hospital of Huaihua, University of South China, Huaihua, 418000 People’s Republic of China; 4 Department of Pharmacology, The Second People’s Hospital of Huaihua City, Huaihua, 418000 People’s Republic of China; 5 Department of Clinical Pharmacology, Xiangya Hospital, Central South University, Changsha, 410008 People’s Republic of China

**Keywords:** Bempedoic acid, Statin, Ezetimibe, Low-density lipoprotein cholesterol, Adverse events, Meta-analysis

## Abstract

**Background:**

Bempedoic acid is a new drug that reduces cholesterol synthesis via inhibiting ATP citrate lyase. It remains unclear whether the combination of bempedoic acid and other lipid-lowering drugs is better than these drugs alone. This study systematically reviewed the efficacy and safety of bempedoic acid monotherapy or combination togethers in hypercholesterolemic patients.

**Methods:**

Randomized controlled trials were searched across Medline, Embase, Cochrane library, web of science, etc. The net change scores [least squares mean (LSM) percentage change] in LDL-C level were meta-analyzed using weighted mean difference. The reductions in other lipids including total cholesterol (TC), non-high-density lipoprotein cholesterol (non-HDL-C) and apolipoprotein (ApoB) and high sensitivity C reactive protein (hsCRP) were also assessed. Odds ratio (OR) of the incidence of adverse events (AEs) were calculated to evaluate the safety of bempedoic acid.

**Results:**

A total of 13 trials (4858 participates) were included. Pooled data showed that the combination togethers resulted in greater reductions in LDL-C level than monotherapies (bempedoic acid + statin vs. statin: LSM difference (%), − 18.37, 95% CI, − 20.16 to − 16.57, I^2^ = 0; bempedoic acid + ezetimibe vs. ezetimibe: LSM difference (%), − 18.89, 95% CI, − 29.66 to − 8.13, I^2^ = 87%). But the difference in efficacy between bempedoic acid and ezetimibe was not obvious. Meta-regression analysis showed the treatment duration was a source of heterogeneity (adj R^2^ = 16.92, 95% CI, 0.04 to 0.72). Furthermore, the background therapy of statin before screening decreased the efficacy of bempedoic acid. In addition, bempedoic acid also resulted in a significant reduction in TC, non-HDL-C, ApoB and hsCRP level. The OR of muscle-related AEs by the combination of bempedoic acid and statin was 1.29 (95% CI, 1.00 to 1.67, I^2^ = 0) when compared with statin alone.

**Conclusion:**

This study showed the efficacy of combination togethers were similar but stronger than these drugs alone. Of note, a trend of high risk of muscle-related AEs by the combination of bempedoic acid and statin was observed, though it is not statistically significant, such risk is needed to be confirmed by more trials, because it is important for us to determine which is the better combinative administration for statin-intolerant patients.

## Background

Lipid-lowering therapy mainly targeting LDL-C is an effective way to reduce cardiovascular risk [1]. Current clinical guidelines all recommend statins as the first-class option both for primary and second prevention [2, 3]. However, even having received maximally tolerated statins, there is also a high proportion of patients hardly reach the LDL-C goal.

Statins inhibit 3-hydroxy-3-methylglutaryl–coenzyme A reductase (HMGCR) to reduce cholesterol-biosynthesis, and thus upregulate the expression of hepatic LDL receptor (LDLR), eventually lead to an increase in LDL-C clearance. Adenosine triphosphate-citrate lyase (ACL), a key enzyme that locates in the upstream of HMGCR in the cholesterol–biosynthesis pathway [4]. It implicates that ACL inhibition may present a similar effect on LDL-C reduction as HMGCR inhibition. Recently a mendelian randomization study reported that variants in *ACLY,* the gene that encodes the ACL, was significantly associated with decreased LDL-C level, as well as decreased cardiovascular risk [5]. These findings provide substantial evidence that ACL could be a threptic target. However, genetic variants result in a lifelong inhibition of ACL, which is much different from the relative shorter-term exposure of ACL inhibitors [6]. Thus, it is necessary to assess the efficacy and safety of ACL inhibitors by using randomized controlled trials (RCTs). Bempedoic acid (ETC-1002), an oral inhibitor of ACL, converts to the active form ETC-1002-CoA by the enzyme very long-chain acyl-CoA synthetase 1 (ACSVL1) [7], which is particularly expressed in the liver but not in the peripheral tissues, including muscles. So, such liver-specific action of bempedoic acid may avoid or decrease the statin-related muscle disorders [8, 9].

Importantly, the clinical use of bempedoic acid is deserved to be explored. With the similar mechanism of action as statins, bempedoic acid is designed to be used in the patients with statin-intolerance. Several meta-analyses reported the efficacy of LDL-C reduction by bempedoic acid [10–12]. However, the biggest uncertainty is whether the combination togethers of bempedoic acid and other lipid-lowering drugs are better than these drugs alone. The current study systematically reviewed the efficacy and safety of bempedoic acid alone or combining with statins or ezetimibe on hypercholesterolemic patients by meta-analyses.

## Methods

This study pooled the trial-level data and followed the principles recommended by the Cochrane handbook for performing and reporting intervention system review [13].

### Data sources and search strategy

Studies regarding to the bempedoic acid treatment in hypercholesterolemia patients were considered as potential eligible. Two investigators (SZH and DZW) independently conducted the literature search through those following databases: Medline, Embase, Cochrane Central Register of Controlled Trials (CENTRAL), web of science and websites (www.clinicaltrials.gov). Two major ways were used to search literature. First, the following keywords (“ETC-1002” OR “bempedoic acid”) AND (“randomized controlled trial” OR “controlled clinical trial” OR “trial”) were used for searching through databases; second, we also performed a manual searching by scrutinizing the reference lists from all relevant articles. Literature search was updated on 11 July 2020. The details of search algorithm of Medline (Via PubMed) were provided in Additional file 1.

### Study selection and data extraction

Predefined inclusion criteria for study selection was listed as follows: (i) study population included adults with hypercholesterolemia received bempedoic acid alone or combining with other lipid-lowering drugs; (ii) end points included efficacy outcomes (the net change scores of lipids level from baseline) and safety outcomes (the incidence of adverse events related to therapy drugs); (iii) the study was designed as clinical trials of randomized, double-blind, placebo- or active-controlled. Of note, studies that missed important information or reported the same population were excluded.

The following information was extracted from the primary text of individual study: the first author, publication year, clinical trial number, follow-up duration, the sample size of randomized patients, demographic and clinical characteristics of participates, baseline LDL-C level, least squares mean (LSM) difference of LDL-C level and the number of AEs across different intervention groups.

Two investigators (JYX and XZP) independently conducted the study selection and data extraction according to standard criteria and data sheets. All the disagreements were resolved through consulting with the third investigator (QCF) to reach the final consensus.

### End points

The efficacy of bempedoic acid was assessed using the net percentage change in LDL-C level from baseline over the follow-up duration. The other lipids including total cholesterol (TC), non-high-density lipoprotein cholesterol (non-HDL-C), apolipoprotein (ApoB) and high sensitivity C reactive protein (hsCRP) were also evaluated. For the overall safety assessment, the incidence of any AEs, serious AEs and muscle-related disorders were compared between different intervention groups.

### Bias risk assessment

Cochrane risk of bias assessment tool was used to judge the bias risk of included trials. Two investigators (LX and JSL) independently assessed the allocation sequence generation, allocation concealment, blinding of participants and investigators, completeness of outcome data and selective outcome reporting of individual trial.

### Statistical analysis

For the efficacy assessment, the net change scores which presented as LSM percentage change in lipids level and hsCRP level from baseline were pooled using the DerSimonian-Laird random-effect model. Where the LSM percentage changes of two groups were reported, the net change score was calculated using the following formula: net change score = LSM percentage change of bempedoic acid group – LSM percentage change of control group [14]. Multiple interventions with different dose and different follow-up in one trial were combined to create a single pairwise comparison by using a weighted average [14]. Pooled effect size was represented as weight mean difference (WMD) of net percentage change and 95% confidence interval (CI). In addition, random-effect meta-regression analyses with the restricted maximum likelihood estimation were performed to evaluate whether the magnitude of LDL-C reduction by bemedoic acid was associated with treatment dose and duration. Subgroup-analyses sorted by statin intensity were also conducted. To estimate the safety of bempedoic acid treatment, the incidence of AEs across groups were compared, odd ratio (ORs) with 95% CI was used to describe the effect estimate.

Between-study heterogeneity was quantitatively calculated using the I^2^ index [15]. Four levels were assigned to the heterogeneity assessment according to the value of I^2^ index (0–25%: no heterogeneity; 25–50%: moderate heterogeneity; 50–75%: large heterogeneity; and 75%–to 100%: extreme heterogeneity). Potential publication bias was evaluated by visual inspection of funnel plots for asymmetry [16]. Two-tailed α level of significance was set at 0.05.

All statistical analyses were performed with Review Manager Version 5.3 (The Nordic Cochrane Center, Copenhagen, Denmark) and STATA/SE.12.0 (StataCorp, College station, Texas, USA).

## Results

### Characteristics of included trials

**Fig. 1:**
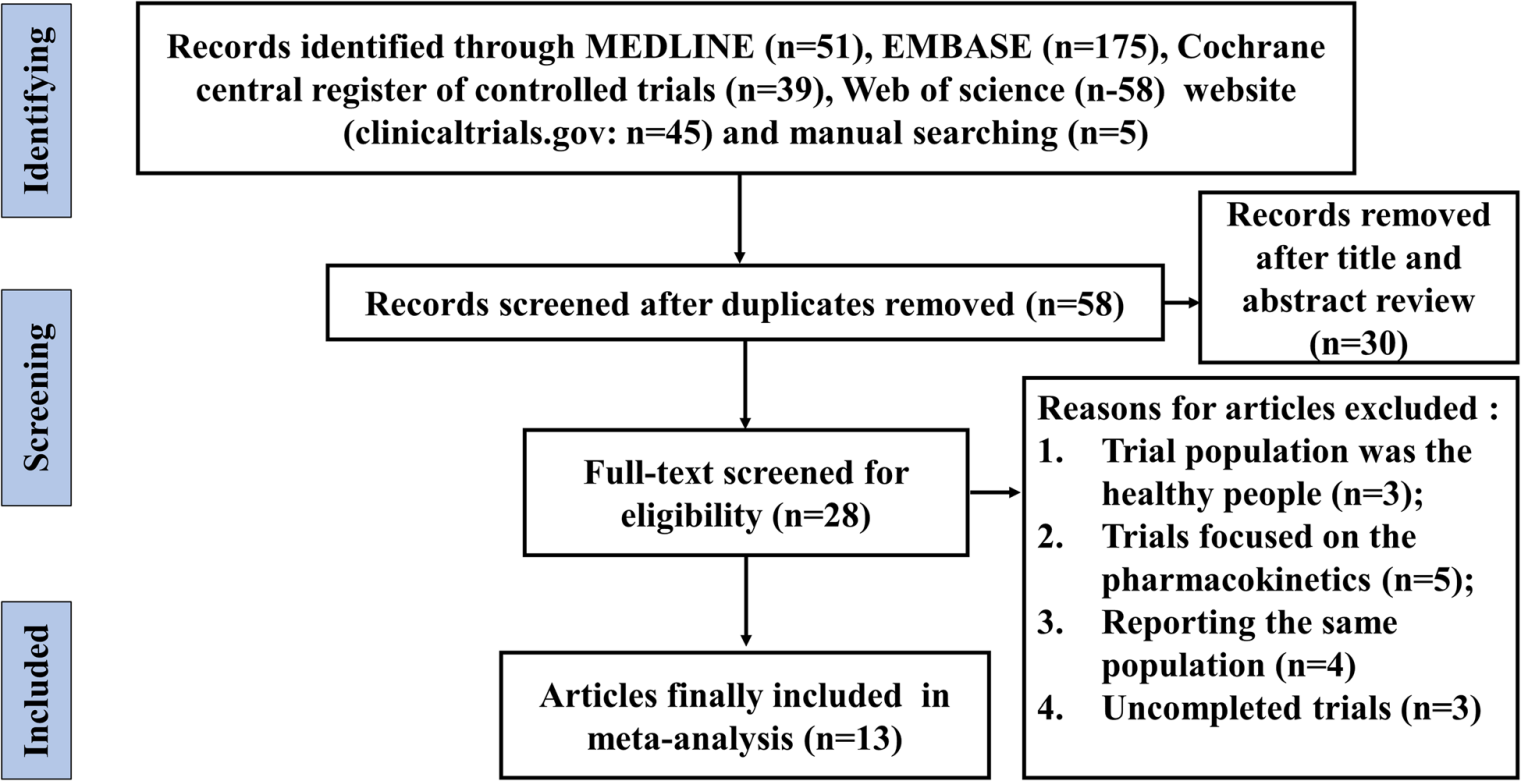
Flow diagram of study screen process

According to the predefined criteria, a total of 13 randomized control trials (RCTs) with 4858 participates were finally included in the current meta-analysis (Fig. 1). Six trials regarded to the combination of bempedoic acid and statins versus statins alone, two trials regarded to the combination of bempedoic acid and ezetimibe versus ezetimibe alone, two trials regarded to the combination of bempedoic acid and ezetimibe versus placebo, five trials regarded to bempedoic acid versus placebo and two trials regarded to bempedoic acid versus ezetimibe. All the participates were hypercholesterolemic patients with the baseline LDL-C level of 70 mg per deciliter at least or more. The mean age of participates was 61 years, 61.82% (3003) were males and 88.18% (4284) were white race. Among them, 72.05% (3500) were at high CVD risk at screening. More details of study characteristics were listed in the Table 1.

**Table 1:** Patient Demographics and Characteristics

Study ID	Follow-up (week)	Randomized patients	Age (mean)	Male no. (%)	White no. (%)	ASCVD risk factor no. (%)	Baseline LDL-C (mean ± SD) (mg/dl)	Arms	LSM Difference (95% CI) percentage points	Total AEs, no (%)	
										BA	Placebo
Ray, K. K. 2019 [17] (NCT02666664)	52	2230 (2:1)	66	1628 (73.0)	2139 (95.9)	2176 (97.6)	103.2 ± 29.4	BA+statin vs. placebo +statin	−18.5 (− 20.7, −16.3)	1167 (78.5)	584 (78.7)
Goldberg, A. C. 2019 [18] (NCT02991118)	52	779 (2:1)	64	496 (63.7)	752 (96.5)	736 (94.5)	120.4 ± 37.9	BA+statin vs. placebo +statin	−17.4 (−21, −13.9)	366 (70.1)	182 (70.8)
Lalwani, N. D.2019 [19] (NCT02659397)	4	68 (2:1)	58	35 (51.5)	49 (72.1)	NA	76.4 ± 22.8	BA+Ato vs. placebo +Ato	−22.2(− 36.4,-8.0)	16 (35.6)	5 (21.7)
Ballantyne, C. M.2019 [20] (NCT03337308)	12	301 (2:2:2:1)	64.4	149 (49.5)	243 (80.7)	188 (62.5)	149.7 ± 41	BA+EZE vs. BA/EZE/placebo	−17.8(−25.1,-10.5)/−12.1(−19.1,-5.0)/− 33.7(− 43.9,-23.4)	53 (62.4)	58 (65.9)/47 (54.7)/18 (43.9)
Laufs, U.2019 (NCT02988115) [21]	24	345 (2:1)	65.2	151 (43.8)	307 (89.0)	NA	157.6 ± 39.9	BA vs. placebo	−18.9(−23.0,-14.9)	150 (64.1)	63 (56.8)
Ballantyne, C. M.2018 [22] (NCT03001076)	12	269 (2:1)	64	104 (38.7)	240 (89.2)	269 (100)	127.6	BA+EZE vs. placebo + EZE	−36.5 (− 45.75, − 27.26)	88 (48.6)	39 (44.8)
McGowan, Mary. 2017 [23] (NCT02659397)	4	68 (2:1)	NA	NA	NA	NA	86	BA+statin vs. placebo +statin	−22 (−36.4, − 8)	NA	NA
Thompson, P. D.2016 [24] (NCT01941836)	12	349	60	244 (70.0)	180 (51.6)	131 (37.8)	164.5 ± 25	BA+EZE vs. placebo + EZE	−26.5 (−32.11, − 20.89)	105 (52.8)	31 (62)
Ballantyne, C. M.2016 [25] (NCT02072161)	12	133 (2:1)	57	54 (40.6)	111 (83.4)	NA	135.4 ± 24	BA+statin vs. placebo +statin	−16.7 (−26.7, − 6.7)	43 (48.9)	28 (62)
Thompson, P. D.2015 [26] (NCT01751984)	8	56 (2:1)	62.6	28 (50)	54 (96.4)	NA	179 ± 35.7	BA vs. placebo	−28.7(−35,4,−22.1)	26 (70)	17 (89)
Newton, Roger S 2014 [27]	8	58	NA	NA	NA	NA	NA	BA+statin vs. placebo +statin	-22 (−32.4, −11.6)	NA	NA
Gutierrez, M. J.2014 [28] (NCT01607294)	4	60 (1:1)	55.7	37 (61.7)	57 (95)	NA	126.8 ± 27.8	BA vs. placebo	−39(−46.2,-31.7)	14 (47)	21 (70)
Ballantyne, C. M.2013 [29] (NCT01262638)	12	177 (1:1:1:1)	57	98 (55.4)	152 (85.9)	NA	166.3 ± 24	BA vs. placebo	−21.1(−26,-16.2)	97 (92.9)	33 (75)

Notes: *BA* Bempedoic acid, *EZE* Ezetimibe, *ASCVD* Arteriosclerotic cardiovascular disease, *FH* Familial hypercholesterolemia, *HC* Hypercholesterolemia, *TC* Total cholesterol, *LDL-C* Low-density lipoprotein cholesterol, *Non-HDL-C* Non-high- density lipoprotein cholesterol, *HDL-C* High density lipoprotein cholesterol, *ApoB* Apolipoprotein, *T2DM* Type 2 diabetes mellitus

### Bempedoic acid significantly reduced LDL-C level

**Fig. 2:**
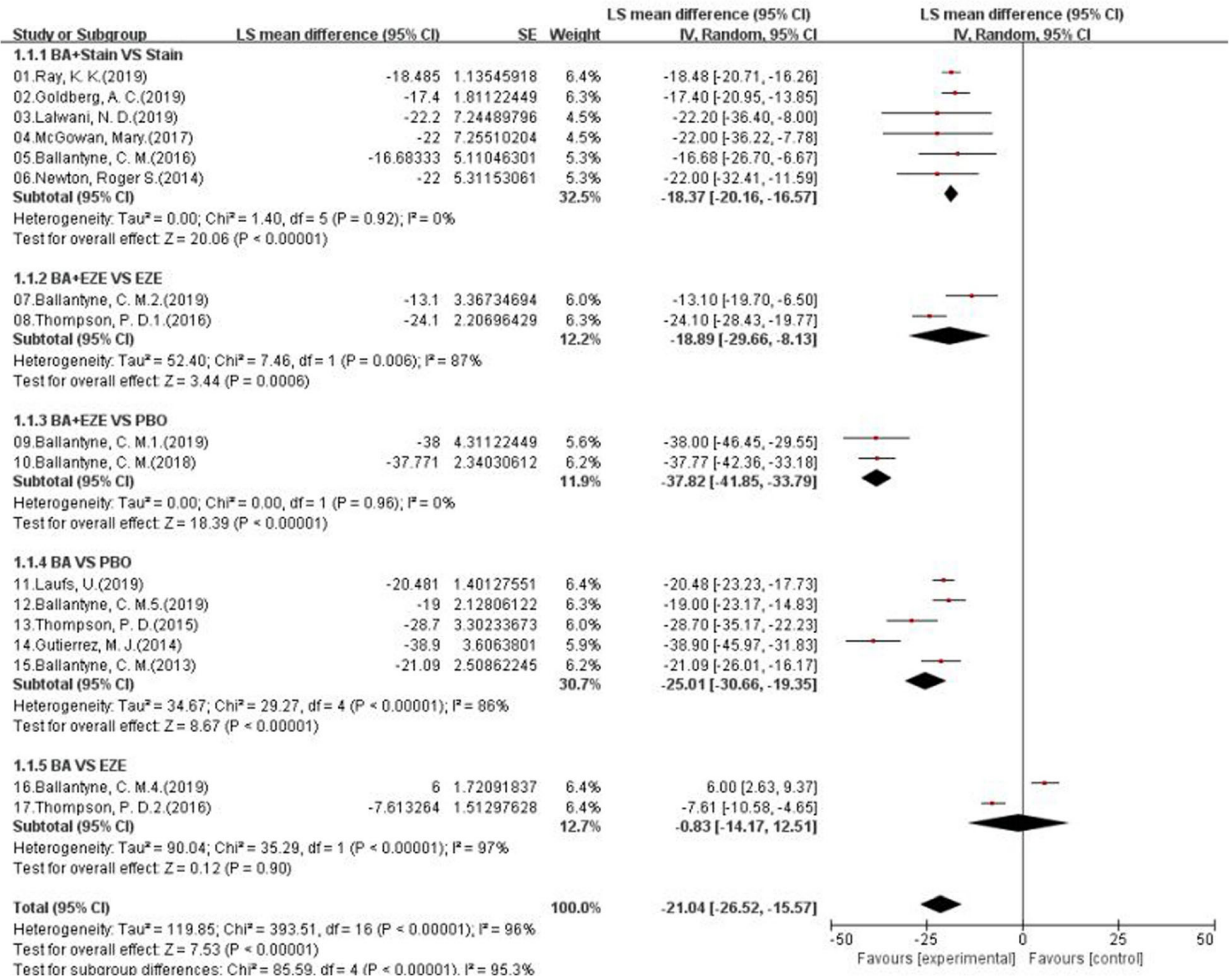
Funnel plot of the reduction in LDL-C level by bempedoic acid

As showed in the Fig. 2, both bempedoic acid monotherapy or combining with statin or ezetimibe were all significantly reduced LDL-C level. Of note, when compared with statin and ezetimibe alone, the combination togethers all resulted in an additional reductions in LDL-C level (bempedoic acid + statin vs. stain, LSM difference [%]: -18.37, 95% CI: − 20.16 to − 16.57, I^2^ = 0; bempedoic acid + ezetimibe vs. ezetimibe, LSM difference [%]: -18.89, 95% CI, − 29.66 to − 8.13, I^2^ = 87%; subgroup differences, *p* = 0.92). The magnitude of LDL-C reduction by the combination together (bempedoic acid + ezetimibe) was greater than bempedoic acid alone (bempedoic acid + ezetimibe vs. placebo, LSM difference [%]: -37.82, 95% CI, − 41.85 to − 33.79, I^2^ = 0; bempedoic acid vs. placebo, LSM difference [%]: -25.01, 95% CI, − 30.66 to − 19.35, I^2^ = 86%; subgroup differences, *p* < 0.000). But difference in the magnitude of LDL-C reduction between bempedoic acid and ezetimibe was not obvious.

**Fig. 3:**
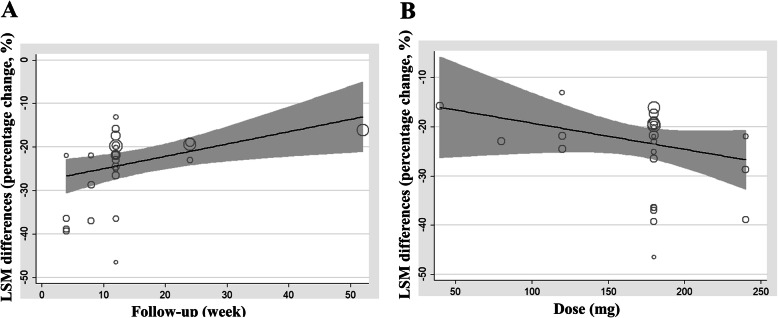
Meta-regression analyses of the influences of treatment duration (**a**) and dose (**b**) on the magnitude of LDL-C-lowering by bempedoic acid

**Fig. 4:**
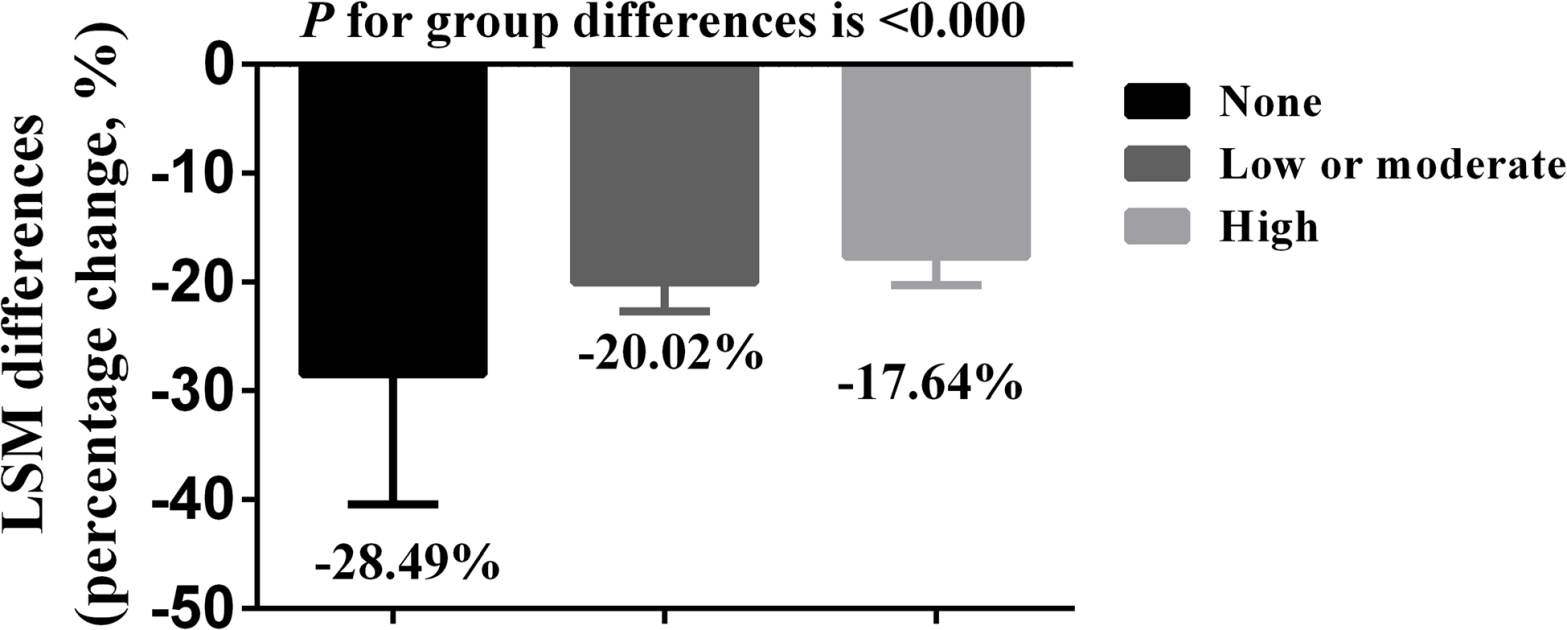
Subgroup-analysis of the LDL-C reductions sorted by the statin therapy and intensity. Data presented as mean ± SE

Meta-regression analysis showed a negative association between the magnitude of LDL-C-lowering and the follow-up duration (Fig. 3 A, adj R^2^ = 16.92, 95% CI, 0.04 to 0.72). Though a trend of positive association between the LDL-C reduction and treatment dose was observed, but it was not statistically significant (Fig. 3 B, adj R^2^ = 9.67, 95% CI, 0.15 to 0.01). When compared with statin alone, the net percent reduction in LDL-C by the combination of bempedoic acid and statin at week 8, 12, 24 and 52 were − 22% (95% CI, − 32.41% to − 11.59%), − 19.13% (95% CI, − 20.80% to − 17.46.%), − 18.65% (95% CI, − 20.57% to − 16.73%) and − 16.10% (95% CI, − 18.35% to − 13.35%), respectively. Furthermore, we conducted a subgroup analysis which stratified by the background therapy of statin before screening, the magnitude of LDL-C reductions across the subgroups represented as − 28.49% (95% CI, − 40.44 to − 16.54), − 20.02% (95% CI, − 22.71 to − 17.34) and − 17.64% (95% CI, − 20.27 to − 15.00) for non-using, low or moderate and high intensity statin group, respectively, *p* value for the differences between groups was < 0.000 (Fig. 4).

### Bempedoic acid reduced TC, non-HDL-C, ApoB and hsCRP levels

Pooled results showed that bempedoic acid also resulted in a significant reduction in TC, non-HDL-C, ApoB and hsCRP levels (Table 2). Compared with the statin alone, the combination of bempedoic acid and statin represented − 11.40% (95% CI, − 12.15 to − 10.64, I^2^ = 32.40%), − 13.57% (95% CI, − 14.52 to − 12.62, I^2^ = 0), 12.73% (95% CI, − 14.71 to − 10.75, I^2^ = 45.80%) and − 20.72% (95% CI, − 29.21, − 12.23, I^2^ = 77.8%) reductions in TC, non-HDL-C, ApoB and hsCRP levels, resepectively. The efficacy of bempedoic acid plus ezetimibe was similar as the bempedoic acid plus statin.

**Table 2:** Pooled results of TC, non-HDL-C, ApoB and hsCRP reductions by bempedoic acid

Subgroups	Number of studies	Sample size		ES (95% CI)	*I*^*2*^(%)
		Experimental	Control		
**TC**					
BA+Stain VS Stain	4	2050	1045	−11.40 (−12.15, −10.64)	2.4
BA+EZE VS EZE	1	86	86	−10.40 (−16.15, −4.65)	–
BA+EZE VS PBO	1	86	41	−27.10(−35.10, −19.10)	–
**Non-HDL-C**					
BA+Stain VS Stain	4	2050	1045	−13.57(−14.52, −12.62)	0
BA+EZE VS EZE	1	86	86	−12.10(−19.15, −5.05)	–
BA+EZE VS PBO	1	86	41	−17.80(−25.10, −10.50)	–
BA VS PBO	1	224	107	−17.10(−20.50, −13.70)	–
**ApoB**					
BA+Stain VS Stain	4	1998	1042	−12.73(−14.71, −10.75)	45.8
BA+EZE VS EZE	1	82	84	−9.30(−16.50.-2.10)	–
BA+EZE VS PBO	1	82	38	−12.80(−20.30, −5.30)	–
BA VS PBO	1	224	107	−15.50(−18.80, −12.20)	–
**hsCRP**					
BA+Stain VS Stain	4	2185	1109	−20.72(−29.21, −12.23)	77.8
BA+EZE VS EZE	1	80	79	−25.60(−44.50, −6.70)	–
BA+EZE VS PBO	1	80	39	−46.10(−77.60, −14.60)	–

Notes: *BA* Bempedoic acid, *EZE* Ezetimibe, *TC* Total cholesterol, *Non-HDL-C* Non-high- density lipoprotein cholesterol, *ApoB* Apolipoprotein, *hsCRP* High sensitivity C reactive protein

### Safety assessment of bempedoic acid treatment

For the safety assessment, bempedoic acid alone or combining with statin or ezetimibe did not show a statistical difference in the incidence of any AEs, serious AEs and the AEs which lead to discontinuation of treatment (Table 3). Of note, the OR of muscle-related AEs by the combination of bempedoic acid and statin was 1.29 (95% CI, 1.00 to 1.67, I^2^ = 0) when compared with statin alone.

**Table 3:** Pooled results of the incidence of AEs by bempedoic acid

Subgroup	Number of studies	Experimental		Control		OR(95% CI)	*I*^*2*^(%)
		AEs	Total	AEs	Total		
**Any AEs**							
BA+EZE VS EZE	3	132	284	101	232	1.26 (0.88,1.84)	0
BA+Stain VS Stain	4	1583	2109	795	1047	0.96 (0.80,1.14)	0
BA+EZE VS PBO	1	13	53	4	18	1.14 (0.32,4.07)	–
BA VS PBO	3	91	217	58	112	0.58 (0.24,1.45)	56.1
BA VS EZE	1	105	198	53	98	0.96 (0.59,1.56)	–
**Serious AEs**							
BA+EZE VS EZE	3	13	284	13	232	0.76 (0.33,1.72)	0
BA+Stain VS Stain	4	323	2109	154	1047	1.05 (0.85,1.30)	0
BA+EZE VS PBO	1	8	53	1	18	3.02 (0.35,26.01)	–
BA VS PBO	2	15	187	4	82	1.53 (0.52,4.53)	0
BA VS EZE	1	3	198	1	98	1.49 (0.15,14.53)	–
**Discontinuation due to AEs**							
BA+EZE VS EZE	3	20	284	23	232	0.70 (0.35,1.39)	0
BA+Stain VS Stain	3	165	1587	56	790	1.12 (0.44,2.89)	29.7
BA+EZE VS PBO	1	7	53	2	18	1.22 (0.23,6.48)	–
BA VS PBO	2	43	187	16	82	0.44 (0.02,9.81)	76.3
BA VS EZE	1	9	198	8	98	0.54 (0.20,1.43)	–
**Muscle-related AEs**							
BA+EZE VS EZE	3	22	284	24	232	0.55 (0.16,1.86)	59.1
BA+Stain VS Stain	4	226	2109	93	1047	1.29 (1.00,1.67)^a^	0
BA+EZE VS PBO	1	6	53	3	18	0.73 (0.23,2.35)	–
BA VS PBO	2	40	187	24	82	0.66 (0.37,1.20)	0
BA VS EZE	1	14	198	12	98	0.55 (0.24,1.23)	–

Notes: *AE* Adverse event, *BA* Bempedoic acid, *EZE* Ezetimibe, *PBO* Placebo. ^a^, statistical significant

### Bias assessment

The allocation sequence generation, blinding of participants and investigators, blinding of participants and investigators and selective outcome reporting were all classified as “low risk of bias” in 13 included trials, allocation concealment in 7 trials and completeness of outcome data in 3 trials were classified as “unclear risk of bias”. The issue of “anything else bias” was also hard to identified in the included trials. (Additional file 2). The potential publication bias of studies which regarded to the comparison of bempedoic acid plus statin versus statin alone was not obvious which was assessed by visual inspection of funnel plots for asymmetry (Additional file 3).

## Discussion

In this study, pooled results showed that both bempedoic acid alone and combining with statin or ezetimibe all significantly reduced LDL-C level, the magnitudes of LDL-C reduction by the combination togethers (bempedoic acid plus statin or ezetimibe) were similar but all stronger than these drugs alone. The treatment duration and the background of statin therapy may be the potential factors that influence the efficacy of bempedoic acid. In addition, bempedoic acid also lead to significant reductions in TC, non-HDL-C, ApoB and hsCRP levels. Importantly, current data showed the combination of bempedoic acid and statin may represent a trend of higher risk of muscle-related disorders when compared with statin alone.

Bempedoic acid, an oral inhibitor, is anticipated to lower the LDL-C level trough blocking ACL [7]. This meta-analysis confirmed that bempedoic acid significantly reduced the LDL-C level, which was consistent with the prior meta-analyses [10–12]. Of note, subgroup-analysis showed that the magnitude of reduction in LDL-C level varied largely among the different types of interventions. Bempedoic acid monotherapy presented the similar efficacy as ezetimibe. Because most participates had a history of statin intolerance before participated in trial, so no trial particularly compared the efficacy of bempedoic acid alone with statin alone. The efficacy of combination togethers (bempedoic acid + statin and bempedoic acid+ ezetimibe) were all stronger than statin or ezetimibe alone. Mechanism action of these agents may interpret the notion of “together is better”. These results support the role of the combinations of bempedoic acid and statin or ezetimibe as an optional lipid-lowering strategy in patients with statin intolerance.

A phenomenon called “trail fatigue” is very common in many drugs studies. It is important to confirm whether the efficacy could be sustained with the extended period of treatment. Current data showed a slight attenuation of LDL-C reduction by bempedoic acid across week 4 to 52 of treatment. Such association needed to be explored in trials with longer-term exposure of bempedoic acid. Bempedoic acid works through the same cholesterol synthesis pathway as statins, it is interesting that whether statin therapy before screening could influence the efficacy of bempedoic acid. We observed the magnitude of reduction in LDL-C level decreased slightly as the growing intensity of statin therapy, but the net change point by bempedoic acid was also obvious even in the patients who have received high intensity of statin therapy, suggesting that bempedoic acid is an alternative for the patients with statin intolerance.

In addition, results showed that bempedoic acid also significantly reduced the other bad lipids level, including TC, non-HDL-C and ApoB, suggesting that bempedoic acid presents an additional effect on lipids level. High hsCRP in patients is closely related to the high risk of cardiovascular events. Clinical trials have demonstrated that patients with high hsCRP at baseline would get the most benefit from the lipid-lowering therapy with statin or PCSK9 inhibitors [30, 31]. Results based on the current data showed that bempedoic acid substantially reduced hsCPR level, indicating that bempedoic acid may result in a benefit for patients in addition to lower lipids.

For the safety analysis, observed differences in any AEs, serious AEs and AEs which lead to discontinuation of treatment were not significant. For the statin-intolerant patients with hypercholesterolemia, clinical guidelines recommend combination therapy with statin and other non-statin lipid-lowering drugs to reach the target of LDL-C level. Reducing the muscle-related disorders is the major concern for the combinative administration. Particularly, the liver-specific effect of bempedoic acid may resulted in reduced risk in muscle disorders as compared with statins. However, pooled results from this study showed a trend of higher risk in muscle-related AEs by the combination of bempedoic acid and statin when compared with statin monotherapy. Though it is not statistically significant, such risk need to be confirmed with more trials, because it is important for us to identify whether the combination of bempedoic acid and statin is safe for clinical practice. However, we did not compare the differences of muscle disorders risk between bempedoic acid alone and stain alone, which is a limitation in evaluating the safety of bempedoic.

Some limitations of this study listed are as follows. First, this study is a trial-level pooled analysis, it is hard to avoid potential bias. Second, though the efficacy of LDL-C reduction has been confirmed, this study failed to identify the cardiovascular risk reduction by bempedoic acid, one trial (CLEAR Outcomes, NCT02993406) is ongoing to assess the absolute CVD benefit of bempedoic acid. Third, the follow-up duration of completed trials was not long enough to observe the occurrence of AEs, which makes it difficult to fully confirm the safety of bempedoic aicd.

## Conclusions

This study showed the efficacy of combination togethers (bempedoic acid plus statin or ezetimibe) were similar but all stronger than these drugs alone. Of note, a trend of high risk in muscle-related AEs by the combination of bempedoic acid and statin were observed, though it is not statistically significant, such risk was needed to be confirmed by more trials, because it is important for us to determine which is the better combinative administration for statin-intolerant patients. Now the absolute cardiovascular benefit of patients from bempedoic acid treatment is unclear, we anticipate the cardiovascular outcomes trials with bempedoic acid give us the answers.

## Supplementary Information

**Additional file 1.** Search algorithm from Medline.

**Additional file 2.** Risk of bias in the included trials as assessed by the Cochrane risk of bias assessment tool.

**Additional file 3.** Publication bias assessment of included trials in the efficacy analysis.

## Data Availability

Data are available from the authors on request.

## Abbreviations

LDL-C: Low-density lipoprotein cholesterol
CVDs: Cardiovascular diseases
AEs: Adverse events
ACL: Adenosine triphosphate-citrate lyase
HMGCR: 3-hydroxy-3-methylglutaryl–coenzyme A reductase
LDLR: LDL receptor
ACSVL1: Long-chain acyl-CoA synthetase 1
RCTs: Randomized control trials
TC: Total cholesterol
Non-HDL-C: Non-high-density lipoprotein cholesterol
ApoB: Apolipoprotein
hsCRP: High sensitivity C reactive protein
LSM: Least squares mean
WMD: Weight mean difference
CI: Confidence interval
RRs: Risk rations
