# Supplementary material for: Efficacy and safety of bempedoic acid alone or combining with other lipid-lowering therapies in hypercholesterolemic patients: a meta-analysis of randomized controlled trials
Source: BMC Pharmacol Toxicol. 2020 Dec 4;21:86. doi: 10.1186/s40360-020-00463-w (PMC7716459; doi:10.1186/s40360-020-00463-w)
Supplement: Supplementary file 1 — Additional file 1. Search algorithm from Medline. [file 40360_2020_463_MOESM1_ESM.docx]

**Additional file 2.** Search algorithm of Medline

| PubMed (Medline) | | |
| --- | --- | --- |
| #1 | Bempedoic Acid | 75 |
| #2 | ETC-1002 | 48 |
| #3 | #1 OR #2 | 78 |
| #4 | randomized controlled trial [Publication Type] | 669,713 |
| #5 | controlled clinical trial [Publication Type] | 738,122 |
| #6 | randomized [tiab] | 1,203,401 |
| #7 | randomly [tiab] | 336,613 |
| #8 | trial [tiab] | 1,725,255 |
| #9 | #4 OR #5 OR #6 OR #7 OR #8 | 2,258,763 |
| #10 | (animals [mh] NOT humans [mh]) | 4,716,501 |
| #11 | #3 AND #9 | 51 |
| #12 | #11 NOT #10 | 51 |
