# Supplementary figures and images for: Efficacy and safety of bempedoic acid alone or combining with other lipid-lowering therapies in hypercholesterolemic patients: a meta-analysis of randomized controlled trials
Source: BMC Pharmacol Toxicol. 2020 Dec 4;21:86. doi: 10.1186/s40360-020-00463-w (PMC7716459; doi:10.1186/s40360-020-00463-w)

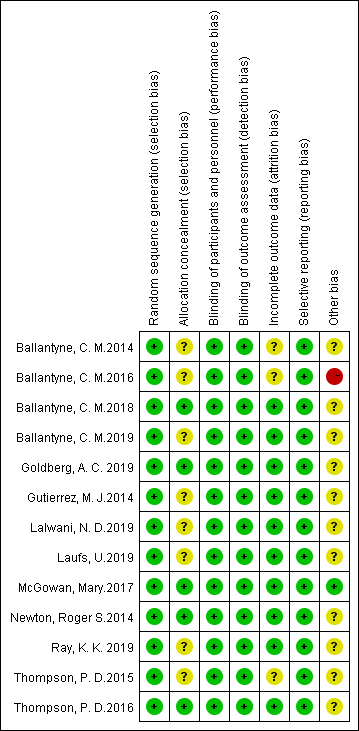

Supplement: Supplementary file 2 — Additional file 2. Risk of bias in the included trials as assessed by the Cochrane risk of bias assessment tool. [file 40360_2020_463_MOESM2_ESM.png]

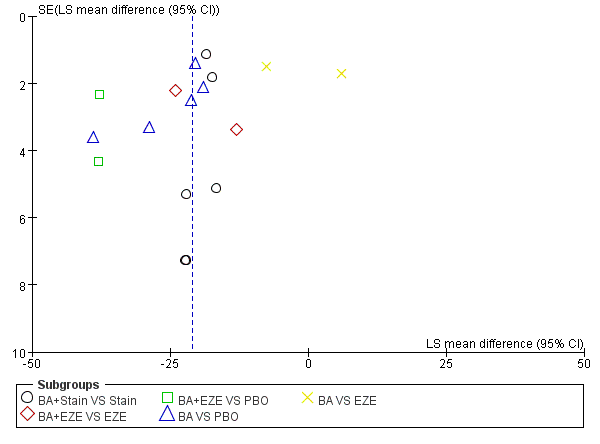

Supplement: Supplementary file 3 — Additional file 3. Publication bias assessment of included trials in the efficacy analysis. [file 40360_2020_463_MOESM3_ESM.png]
